# HPV and coronary diseases in menopausal women: an integrative review

**DOI:** 10.61622/rbgo/2024rbgo57

**Published:** 2024-07-26

**Authors:** Andrea de Neiva Granja, Andressa Bianca Reis Lima, Paulo Victor Brito Martins, Bernardete Jorge Leal Salgado, Rui Miguel Gil da Costa, Haissa Oliveira Brito, Natalino Salgado

**Affiliations:** 1 Universidade Federal do Maranhão São Luís MA Brazil Universidade Federal do Maranhão, São Luís, MA, Brazil.

**Keywords:** Papillomavirus humano, Coronary artery disease, Climacteric, Menopause

## Abstract

High-risk human papillomavirus (HPV) infection is associated with cervical cancer while low-risk HPV strains mostly cause benign lesions. Multiple studies have also associated HPV with coronary artery (CAD) disease in women. Furthermore, the climacteric period in women, triggers chronic inflammation and has major implications for CAD and associated lipid disorders. The association of HPV with coronary artery disease in climacteric women has few studies, and the objective of this review is to gather and analyse scientific data on the subject. This is an integrative review performed on PubMed and Google Scholar using the keywords “HPV”, “coronary heart disease” and “climacteric”, among these keywords the boolean operator AND and the publication date filter. (2018 onwards). Five articles were found, whose main results show presence of high-risk vaginal HPV in climacteric women. Climacterium and HPV were associated with a three-fold increased risk of CAD, as well as with factors related to menopause that promote atheroma formation, lipid disorders and chronic inflammation. Thus, these results support the association between HPV infection and CAD in climacteric women, possibly via chronic inflammation, hormonal factors related to menopause and dyslipidemia.

## Introduction

Cervical cancer ranks 9th in terms of global incidence, with over 600,000 new cases estimated in 2020, and it holds the 4th position when considering only the female population.^(1)^ The etiological agent of cervical cancer is human papillomavirus (HPV) infection.^(2)^ Papillomaviruses are small, non-enveloped viruses with a circular DNA genome that infect numerous animals, including humans.^(3,4)^ Papillomaviruses encode genes that are expressed early in the viral cycle (referred to as early genes, or E genes) and genes that are expressed late (late genes, or L genes). While late genes encode structural proteins of the viral capsid, certain early genes, particularly E6, E7, and E5, encode oncoproteins capable of interfering with essential cellular functions such as proliferation and survival.^(5)^ The development of cervical carcinoma is associated with high-risk HPV strains (HR-HPV) that are capable of promoting the integration of viral DNA into the genome of host epithelial cells, resulting in uncontrolled expression of E6 and E7 viral genes. This phenomenon promotes cellular transformation and the potential subsequent progression to an invasive stage of the disease.^(6,7)^ In addition to cervical cancer, clinical and experimental studies have shown that HPV causes multiple other anogenital cancers, such as anal, vulvar, vaginal, and penile cancer. Furthermore, an important and increasing proportion of oropharyngeal cancers are also attributed to HPV.^(5,8-11)^

Although HPV infects keratinized epithelia of the skin and mucous membranes identified an association between high-risk HPV infection and coronary artery disease in women.^(12)^ These authors, who first described this association, examined data from the National Health and Nutrition Examination Survey in the United States (2003 to 2006) and selected a cohort of 60 women with coronary artery disease and a prior HPV test. Surprisingly, HPV infection was associated with an increased risk of developing coronary artery disease (CAD). This is a highly intriguing observation since if HPV, which is the most common sexually transmitted infection, is a risk factor for the development of CAD, its prevention could lead to significant health benefits beyond the expected prevention of neoplastic lesions.

In parallel, considering the perimenopausal period in women, menopause is associated with the establishment of chronic systemic inflammation and has significant implications for the development of CAD.^(13,14)^and associated lipid disorders.^(15)^ The incidence of HPV infection is also higher among women in the perimenopausal and menopausal stages,^(16)^ and it may act synergistically with some of these inflammatory and metabolic factors to exacerbate cardiovascular risk in this female population.

However, this association between HPV infection and CAD in perimenopausal women needs further investigation. Therefore, the aim of this study is to gather and analyze existing information in the scientific literature on this topic through an integrative review.

## Methods

This study is an integrative review based on scientific articles retrieved from the PubMed and Google Scholar bibliographic databases. The following keywords were used: “HPV,” “coronary heart disease,” and “climacteric,” with the Boolean operator “AND” between them. Additionally, a publication date filter was applied (from January 2018 to December 2022). All types of scientific articles were included in the analysis, including reviews, original articles, and editorials. On the other hand, articles published before the year 2018 and those that did not encompass the keywords were excluded. The initial analysis of the literature proceeded as follows: abstracts were read, and when necessary, the introduction and results sections were reviewed to determine if the initially selected articles met the inclusion criteria or fell within the exclusion criteria. Subsequently, the selected articles were organized into tables, including information such as the year, authors, title, study type, and key findings. This integrative review method allows for a comprehensive overview of complex concepts, theories, or health care-related issues by encompassing original and review studies.

## Results

Through the literature review, 5 articles were found and selected from the Pubmed and Google Scholar databases. The data from these articles are presented in chart 1.

**Chart 1:** Data of the selected articles for the study

Authors	Title	Year	Study Type
Brito et al.^(17)^	*Human papillomavirus and coronary artery disease in climacteric women: is there an association?*	2019	Cross-sectional study
Reis et al.^(18)^	*HPV infection as a risk factor for atherosclerosis: a connecting hypothesis.*	2020	Review article
Carvalho et al.^(19)^	*The impact beyond cancer of the HPV vaccine*	2020	Letter to the editor
Tonhajzerova et al.^(20)^	*Novel biomarkers of early atherosclerotic changes for personalised prevention of cardiovascular disease in cervical cancer and human papillomavirus infection.*	2019	Review article
Machado de Carvalho et al.^(21)^	*Prevalence of Dyslipidemia in HIV-Positive Women with HPV Coinfection: A Preliminary Study*	2021	Clinical, cross-sectional

In the pioneering study on this topic in Brazil, the authors observed a statistically significant association between the presence of high-risk HPV DNA at the cervicovaginal level in menopausal women with CAD, after adjusting for age and ethnicity (OR = 4.90, p = 0.0384)^(17)^ It is worth noting that the presence of other types of HPV, low-risk strains, was not associated with CAD in this study, suggesting that high-risk HPV types have specific mechanisms that contribute to the risk of CAD. This observation may facilitate the identification of these mechanisms by investigating the molecular and pathophysiological differences between high and low-risk HPV. The same study revealed that 44.2% of all women studied tested positive for HPV DNA, and 69.6% of these women had CAD. Therefore, the authors support the hypothesis that HPV infection associated with CAD occurs due to a high-risk HPV type, suggesting that the virus affects coronary arteries, promoting the development of atherosclerotic plaques. It was also suggested that HPV may promote the development of atheroma plaques through its classic cellular targets, the p53 protein and the retinoblastoma protein (pRb), as loss of p53 protein function in macrophages has been strongly associated with increased atherosclerotic lesions.^(22,23)^ Further studies involving larger populations would be useful to confirm the findings of this study.

## Discussion

In the following study, hypotheses were presented to justify the association between HPV and CAD.^(18)^ It was suggested that HPV infection may promote the development of atheromas in several ways, either by increasing systemic inflammation and consequent endothelial damage or by HPV nucleic acids and proteins reaching the endothelial cells in the blood vessel walls through the bloodstream, for example, via extracellular vesicles such as exosomes. This latter hypothesis would explain observations from a previous study, which described that women with vaginal HPV have a threefold higher risk of cardiovascular diseases compared to HPV-negative women.^(17)^ In that study, HPV DNA and proteins were detected in 50% of atherosclerotic coronary arteries in a small sample of 20 deceased donors.^(22)^

Moreover, occasional studies claim that HPV can be found in circulating leukocytes in the blood^(24,25)^ and endothelial cells,^(26)^ which may help localize the virus to atherosclerotic plaques. Interestingly, the type of HPV observed in endothelial cells and blood leukocytes in these studies^(25,26)^ was also the high-risk HPV16, which has been associated with an increased risk of CAD. The presence of HPV in these cell types is uncommon, but some animal papillomaviruses are also known to infect other cell types beyond the basal keratinocytes of keratinized epithelia.^(4)^

Another line of thought suggests that HPV, like other viruses, interferes with lipid metabolism, which may also contribute to CAD. In another study, the findings demonstrate a significant association between HPV infection and coronary artery disease (CAD) among menopausal women, especially HPV19.^(19)^ In this study, the HPV-positive group had lower blood levels of HDL cholesterol and higher blood pressure compared to the HPV-negative group, which could be explained by a possible association between HPV infection and lipid metabolism. Furthermore, in the study by Machado de Carvalho et al.,^(21)^ which included 82 HIV-positive women, predominantly aged 35 to 49 years, it was observed that 50% of the patients were also positive for HPV DNA. Regarding risk factors for CAD, such as dyslipidemia, the most common comorbidities in women with HIV and HPV coinfection were dyslipidemia (46.3%) and smoking (46.3%), unlike the group without coinfection. Although HPV is known to alter cellular lipid metabolism,^(27)^ additional studies are needed to clarify its potential role in regulating systemic lipid metabolism.

In addition to the regulation of lipid metabolism, endogenous sex hormones (estrogens, progesterone) in premenopausal women have shown cardioprotective effects, resulting in a lower incidence of cardiovascular diseases. Since the 20th century, it has been suggested that sex hormones, such as estrogens, appear to be a necessary factor for the progression of vaginal HPV infection to malignant disease,^(28)^ and increased exposure to sex hormones, for example, due to prolonged use of oral contraceptives, is associated with an increased risk of cervical cancer.^(29)^ On the other hand, Kuo and Fujise^(12)^suggest that HPV infection may somehow cooperate with factors related to menopause (e.g., hypoestrogenism, adipose tissue remodeling) to promote atheroma formation.

Although HPV has mechanisms that allow it to evade immune system surveillance, notably by minimizing local inflammation, advanced lesions can induce significant inflammatory phenomena.^(30)^ In fact, it has been observed that older women with persistent HPV infection of the uterine cervix showed elevated levels of pro-inflammatory cytokines.^(29)^ Theoretically, this increase in pro-inflammatory factors may also contribute to a higher risk of CAD, but there is currently no data to support this possibility.

Finally, low socioeconomic status is a well-known risk factor for a wide range of diseases, including cervical cancer and cardiovascular diseases, particularly those related to atherosclerosis.^(29-33)^ Improving the living conditions of disadvantaged populations, their level of education, and access to healthcare, including vaccination programs, are crucial steps in promoting health.

## Conclusion

A study reported a significant association between HPV infection and CAD in menopausal women. It is important to note that high-risk HPV genotypes associated with cancer were selectively associated with CAD, while no association was observed with low-risk genotypes. This is consistent with previous studies that detected the two common high-risk HPV types, HPV16 and HPV18, in atherosclerotic plaques, but not low-risk types. Thus, the review supports the hypothesis that there is an association between HPV infection and coronary artery disease in menopausal women. Hypotheses regarding the role of HPV in atherogenesis include increased levels of circulating inflammatory mediators, expression of oncogenic proteins E6 and E7 in cells involved in atherogenesis, and an influence on lipid metabolism. Further studies using larger populations and experimental models are needed to establish and better elucidate the role of HPV in atherogenesis.
